# Two Different Conformations in Hepatitis C Virus p7 Protein Account for Proton Transport and Dye Release

**DOI:** 10.1371/journal.pone.0078494

**Published:** 2014-01-07

**Authors:** Siok Wan Gan, Wahyu Surya, Ardcharaporn Vararattanavech, Jaume Torres

**Affiliations:** School of Biological Sciences, Nanyang Technological University, Singapore, Singapore; Rosalind Franklin University of Medicine and Science, United States of America

## Abstract

The p7 protein from the hepatitis C virus (HCV) is a 63 amino acid long polypeptide that is essential for replication, and is involved in protein trafficking and proton transport. Therefore, p7 is a possible target for antivirals. The consensus model for the channel formed by p7 protein is a hexameric or heptameric oligomer of α-helical hairpin monomers, each having two transmembrane domains, TM1 and TM2, where the N-terminal TM1 would face the lumen of this channel. A reported high-throughput functional assay to search for p7 channel inhibitors is based on carboxyfluorescein (CF) release from liposomes after p7 addition. However, the rationale for the dual ability of p7 to serve as an ion or proton channel in the infected cell, and to permeabilize membranes to large molecules like CF is not clear. We have recreated both activities *in vitro*, examining the conformation present in these assays using infrared spectroscopy. Our results indicate that an α-helical form of p7, which can transport protons, is not able to elicit CF release. In contrast, membrane permeabilization to CF is observed when p7 contains a high percentage of β-structure, or when using a C-terminal fragment of p7, encompassing TM2. We propose that the reported inhibitory effect of some small compounds, e.g., rimantadine, on both CF release and proton transport can be explained via binding to the membrane-inserted C-terminal half of p7, increasing its rigidity, in a similar way to the influenza A M2-rimantadine interaction.

## Introduction

Hepatitis C virus (HCV) has chronically infected about 170 million people worldwide and no prophylactic or therapeutic vaccine is available. The p7 protein, encoded by HCV, is a small transmembrane (TM) protein 63 residues long and is found mainly at the endoplasmic reticulum (ER) membrane. Although p7 is not necessary for RNA replication [1], it is essential for infectivity, assembly, and release of infectious virions [2], [3].

Oligomers formed by p7 show cation-selective channel activity [4]–[6] which is blocked in some cases by amantadine, rimantadine, hexamethylene amiloride (HMA) and long-alkyl-chain imino sugar derivatives [4]–[7]. Recently, membrane permeabilization to protons was found to be crucial for the production of infectious viruses, and was observed in intracellular vesicles harboring p7 [8]. Additionally, p7 is involved in the capsid assembly and envelopment and localization of several viral proteins, possibly via channel-independent mechanisms [9]–[11].

The p7 protein is predicted to have two α-helical TM domains, but details on its three dimensional structure are limited. Solution NMR data has been obtained for synthetic p7 (C27A mutant) in 50% TFE [12], a helix inducer, and for recombinant p7 (C27S mutant) in DHPC micelles [13]. Both papers report a similar percentage of α-helix, 60–70%, and predict an α-helical hairpin with two α-helical TMs kinked in the middle [12], [14], where the N-terminal TM helix (TM1) would face the lumen of a channel [15]–[17] formed by either six or seven monomers [4], [12], [18], [19]. Recently, a model formed mostly of α-helical domains has been reported in DPC micelles and electron microscopy [20].

Alternative topologies and conformations of p7 have been reported in the literature. For example, using ectodomain CD4 or myc epitope tags [15] both N- and C-termini of p7 were exposed extracellularly, consistent with an α-helical hairpin formed by two TM domains. However, a non-tagged p7 showed cytoplasmic orientation for its C-terminus, compatible with a form with only one TM domain [21], [22]. More recently, two very different CD spectra (SRCD), one consistent with an α-helical form, and another that was not assigned to any particular structure, both showing some channel activity, were obtained when p7 protein was reconstituted in membranes with varying PC∶PE ratios [23]. Overall, the above data indicate that the topology and conformation of p7 may not be unique.

In addition to its ion and proton channel activity, p7 has also been shown previously to permeabilize liposomes inducing release of carboxyfluorescein (CF) when added to CF-loaded liposomes [7], [24]. The claimed sensitivity of this procedure to inhibitors such as rimantadine, which also has been proposed by some authors to inhibit p7 ion channel activity, has resulted in this method being proposed as a functional assay to test p7 mutants or discover p7 channel activity inhibitors [25], [26].

However, the presumed dual ability of an oligomer formed by α-helical hairpins to serve both as a channel for small ions or protons, and as facilitator for large molecules like CF, is puzzling. Thus, we hypothesized that the conformation of p7 in these two cases is not the same. Yet, that both activities can be inhibited by similar small molecules indicates that the binding mode of these drugs to p7 in the two instances must be similar, and that inhibition of the two activities must be based on a similar mechanism.

Thus, to establish a correlation between p7 conformation and its diverse activities, we have studied the effect of solvents and type of reconstitution on the structural features of p7 protein. We have used attenuated total reflection Fourier Transform infrared (ATR-FTIR) spectroscopy, a technique that is especially suitable for studies in lipid bilayers. We have used three methods of reconstitution in lipidic membranes. The first two methods co-reconstitute p7, after the protein is dried from a solvent, with lipids, either with the aid of detergent – ‘dialysis’ method – or without detergent – ‘direct’ method –. These two procedures may be followed by a freeze-thawing and extrusion protocol. In the third method, – ‘addition’ method – p7 is added directly from solvent to preformed liposomes, as in the CF-release assay [7], [24]. All of these methods have been used previously in the literature. The ability of p7 to elicit CF release and proton transport has been tested in the controled conditions where the p7 conformation was known, which allowed to infer that the ‘α-helical hairpin’ form is responsible for proton transport, but not of CF release.

## Materials and Methods

### Cloning of p7 gene

The nucleotide sequence corresponding to p7 (HCV subtype 1a, strain H77) was obtained from NCBI (accession number NCBI ID: **ACH61709**). The p7 gene was synthesized and the purity was determined by agarose gel electrophoresis. The p7 gene was cloned into pTBMalE vector with MBP as fusion partner carrying a His-tag at the N-terminus, forming the construct His-MBP-p7. For some of the experiments, an additional FLAG tag was added N-terminally to p7 by insertional mutagenesis using an appropriate set of primers [27], forming the construct His-MBP-FLAG-p7.

### Over-expression of the MBP-p7 construct

The DNA plasmid containing the p7 gene was transformed into an *E. coli* competent cell strain BL-21 (DE3) CodonPlus-RIL (Stratagene) for protein over-expression. Cells from a single colony were picked to inoculate 10 mL LB media with 100 µg/ml ampicillin and 34 µg/ml chloramphenicol, and grown overnight at 37°C with shaking. A volume of 8 mL of the overnight culture was transferred to 800 mL Terrific Broth (TB) media with 1∶100 dilutions and grown at 37°C with shaking to an OD_600_ of 0.6–0.7. The cells were induced with 0.4 mM isopropyl-β-thiogalactoside (IPTG) and grown at 30°C overnight with shaking. For the ^15^N-labeled sample, cells were harvested when an OD_600_ of 0.6–0.7 was reached, and washed with M9 minimal media once. The cells were transferred to M9 minimal media containing ^15^N ammonium chloride. A high cell density strategy was employed by concentrating 4L of culture to 1L media to enhance expression level in minimal media. After induction, cells were harvested and resuspended in Ni^2+^-NTA binding buffer containing 20 mM Tris-HCl, 500 mM NaCl, and 5 mM imidazole, pH 8.0, and then kept frozen at −20°C overnight. Thawed cells were incubated with 0.2 mg/ml lysozyme and 0.02 mg/ml benzonase for 10 min. Then, Triton-X100 was added to the sample to a final concentration of 1%. The cells were lysed with a microfluidizer at 15 kPSI pressure and supernatant was collected after centrifugation at 20,000 g for 30 min and loaded to an Econo column (BioRad) packed with Ni^2+^-NTA agarose resin (QIAGENE) that was pre-equilibrated with binding buffer. The fusion proteins were allowed to bind to the resin with gentle shaking at 4°C, overnight. The nickel resin with bound fusion protein was washed with 20 column volumes of buffer containing 20 mM Tris-HCl, 500 mM NaCl, and 20 mM imidazole, pH 8.0, to remove unbound proteins. The bound proteins were eluted with elution buffer containing 20 mM Tris-HCl, 500 mM NaCl, 500 mM imidazole, pH 8.0, and 5 mM C14-betaine (C14SB). All fractions collected were stored at 4°C. The fractions containing the fusion protein were checked by SDS-PAGE.

### Expression and purification of TEV protease

Expression and purification of TEV protease was done in a similar way for p7 protein except no detergent was added to the sample as it is a soluble protein. TEV was stored at −20°C in buffer containing 50% glycerol, 10 mM Tris-HCl, 250 mM NaCl, 250 mM imidazole, 1 mM EDTA, and 5 mM DTT, pH 8.0.

### Purification of recombinant p7 protein

After purification using Ni^2+^-NTA resin, the construct was subjected to TEV enzymatic cleavage in order to remove MBP. TEV protease was added to the fusion protein at a mass ratio of 1∶5 (TEV: fusion protein). The digestion was performed at room temperature with gentle shaking, and the progress of the reaction was monitored by SDS-PAGE. The digestion was stopped by addition of trichloroacetic acid (TCA) at a final concentration of 6% in volume, and the precipitate was collected by centrifugation at 18,000 g for 30 min. The pellet was washed with water twice followed by lyophilization. p7 was extracted by methanol (10 ml methanol per 1L culture), mixing gently for 2 hours at room temperature. After removal of the insoluble fraction by centrifugation at 18,000 g for 30 min, the supernatant, contained mostly p7 protein. The p7 protein was further purified by injecting the supernatant onto a Zorbax C3-300 Å column connected to HPLC system. The p7 protein was eluted with a linear gradient of solvent A (water/TFA, 99.9∶0.1, v/v) and solvent B (isopropanol/acetonitrile/TFA, 80∶19.9∶0.1, v/v/v). Pooled fractions were lyophilized and the purity was assessed by mass spectrometry. The TEV cleavage results in additional N-terminal SNA residues, as described [28]. An N-terminal methionine was also included as an alternative cleavage point with CNBr, in case the enzymatic cleavage was not successful. The channel activity of p7 protein was tested in black lipid membranes (Fig. S1 in File S1).

### Peptide synthesis and purification

The synthetic peptides p7_1-63_, p7_1-26_ and p7_27-63_ were obtained by solid-phase synthesis using FMOC chemistry. Synthetic p7_1-63_ was purified by RP-HPLC as described above for recombinant p7. The p7 protein from hepatitis C virus (HCV) used here has 63 amino acids [6]. Fragment p7_1-26_ was synthesized manually on a 4-methyl-benzhydrylamine (MBHA) resin using standard *tert*-butoxycarbonyl (Boc) chemistry. The thioester group was incorporated in the resin before chain elongation. Deprotection was done in 30% trifluoroacetic acid (TFA). To monitor each coupling and deprotection step, we used a ninhydrin test. Final peptide cleavage from the resin was achieved by hydrogen fluoride (HF). Fragment p7_27-63_ was synthesized by microwave assisted solid phase fluorenylmethyloxycarbonyl (FMOC) chemistry using an Odyssey microwave peptide synthesizer (CEM Corporation). Both fragments were purified by RP-HPLC on a C4-semipreparative column with a linear acetonitrile gradient and purity was checked by SDS electrophoresis (Fig. S2 in File S1) and MALDI (Fig. S3 in File S1). The two peptides were ligated in 8 M urea, 48 mM DPC, 20 mM TCEP, 90 mM MESNA (catalyzer), and 20 mM phosphate buffer at pH 8.0. After 36 h, the HPLC chromatogram showed fragment A consumed by the reaction, and SDS electrophoresis shows the band corresponding to full length p7 after 5 h of reaction (Fig. S4 in File S1). The MALDI mass spectrum of the reaction mixture (Fig. S5 in File S1) shows the presence of the ligated p7 polypeptide.

### Gel electrophoresis

Standard SDS-PAGE was performed with DTT in 15% Tris-Glycine gel with TGS running buffer or in NuPAGE under non-reducing conditions (4–12% Bis-Tris gel with MES-SDS running buffer) according to the manufacturer's protocol (Invitrogen). The sample, 5 µg, (final concentration of 2 µg/µl) was mixed with sample buffer for 1 min followed by heating at 95°C for 5 min before loading into the gel. The gel was run at constant voltage of 200 V for 50 min at room temperature. The standard SDS-PAGE gels were stained with Coomassie blue while the NuPAGE gels were stained with SimplyBlue SafeStain (Invitrogen) or silver stain (Bio-Rad).

### Infrared spectroscopy

ATR-FTIR spectra were recorded as described previously [29]. Approximately 100 µl of sample in water with 50∶1 lipid/protein molar ratio were applied onto a trapezoidal (50 mm×2 mm×20 mm) Ge internal reflection element (IRE). The area of the amide I (C = O stretching) were obtained by peak integration from 1600 to 1700 cm^−1^, and the area of amide II (N-H bending, centered at ∼1550 cm^−1^) were obtained by peak integration from 1510 cm^−1^ to 1580 cm^−1^. No difference in band area was observed employing other means of peak size estimation such as peak fitting and Fourier self-deconvolution. For the hydrogen-deuterium exchange experiment, the exchange was calculated by measuring the relative area of amide II relative to amide I band, before and after addition of D_2_O, according to Equation 1.
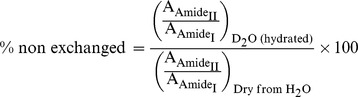

**Equation 1.** Formula to calculate the percentage of non-exchanged amino acids, as described previously [29].

### p7 reconstitution in lipids

We used three methods for p7 reconstitution, which started from recombinant or synthetic p7 samples solubilized in a suitable solvent, e.g., methanol or HFIP. In the first ‘dialysis’ method, the solubilized p7 in organic solvent was first dried under N_2_ gas, followed by overnight lyophilization. Reconstitution of p7 into DMPC lipid bilayers was performed by solubilizing the protein in 30 mM 1,2-dihexanoyl-sn-glycero-3-phosphocholine (DHPC), followed by mixing with DMPC lipids. The mixture was left at room temperature for 1 h, followed by detergent removal with Biobeads (BioRad). This last step was repeated three times in order to completely remove the detergent. If indicated, the mixture was then extruded through a 400 nm pore size membrane.

In the second ‘direct’ method, this solution was mixed with a chloroform solution containing 1,2-dimyristoyl-sn-glycero-3-phosphocholine (DMPC) (Avanti Polar Lipids). The mixture was dried to remove all solvent, and the dry residue was hydrated with 20 mM phosphate buffer pH 7.0 to form the liposomes. Where indicated, the suspension was subsequently vortexed, freeze-thawed 3 times and extruded through a 400 nm pore size membrane. Other than DMPC, 1,2-dioleoyl-sn-glycero-3-phosphocholine (DOPC) – 1-palmitoyl-2-oleoyl-sn-glycero-3-phosphocholine (POPE) lipid mixtures at 1∶4 and 4∶1 w/w ratio have also been used.

In the third ‘addition’ method, liposomes were extruded first with a 400 nm pore size membrane to get small unilamellar liposomes. The protein stock in organic solvent (∼10 mg/ml solution) was added to the liposome solution to a protein to lipid molar ratio of 1∶50.

### Liposome carboxyfluorescein (CF) release assay

Two variants of the liposome CF release assay were performed. In the first case (a), p7 was added to preformed CF-loaded liposomes. An appropriate volume (1 mL) of buffer (60 mM KH_2_PO_4_, 60 mM K_2_HPO_4_, 75 mM NaCl and 10 mM KCl, pH 7.0) containing carboxyfluorescein (CF) at self-quenching concentration (50 mM) was used to re-hydrate dry lipid at 5 mg/ml. This was followed by three freeze-thawing cycles and extrusion through a 0.2 µm membrane filter to produce unilamellar liposomes. Non-incorporated CF dye was removed using an Econo-Pac® 10 DG column (Biorad). To initiate the liposome CF release assay, the liposome solution was diluted 5× with the buffer above and aliquoted to a microtiter plate (100 µL/well). Typically 5 µg of protein, dissolved in either 1 µl HFIP (1% final volume) or 2.5 µl methanol (2.5% final volume), were added to the liposome solution. Fluorescence values were read every minute for ∼40 min. Rimantadine was prepared in ethanol, 1% v/v, and added to the liposome solution at a final concentration of up to 100 µM. In the second case (b), p7 was added to the dried lipid prior to hydration. The protein-lipid suspension was subsequently freeze-thawed, extruded, and separated from non-incorporated CF in the same manner described above. Fluorescence values were read immediately, and after one hour. Fluorescence measurements were performed in a TECAN Microplate Reader (Model Infinite M200 Pro) with excitation and emission at 485 nm and 520 nm, respectively. Baseline fluorescence was determined with samples identical as those described; except that no protein was added. Addition of Triton X-100 to a final concentration of 1% (v/v) was used as a positive control for CF release. Melittin from honey bee venom, also used as a control, was purchased from Sigma-Aldrich and used without further treatment.

### Liposome based proton transport assay

The p7 polypeptide was solubilized in either HFIP or methanol prior mixing with *E. coli* lipids (Avanti Polar Lipids) or a lipid mixture PAESC, i.e., PA/PE/PS/PC (5∶2∶2∶1 w/w), in chloroform at protein to lipid molar ratio 1∶125. The mixture was dried with nitrogen gas. Liposomes were formed with a strong buffer (60 mM KH_2_PO_4_, 60 mM K_2_HPO_4_, 150 mM NaCl and 20 mM KCl, pH 7.0) to a lipid concentration of 10 mg/ml, and extruded 21 times through 0.2 µm polycarbonate membranes. The external solution was exchanged to a weaker buffer (190 mM Na_2_SO_4_, 0.1 mM KH_2_PO_4_ and 0.1 mM K_2_HPO_4_, pH 7.0) over an Econo-Pac® 10 DG column (Biorad). The column was pre-equilibrated with weakly buffered external buffer and 2 ml of the same buffer was used to elute the liposome fraction from the column. The liposome fraction was then further diluted with weak external buffer to approximately 1–2 mg/ml prior the assay.

A highly sensitive pH probe (Mettler Toledo InLab®Micro) was placed in the experimental cuvette containing 3 mL of liposome solution in weak buffer. The experiment was initiated by lowering the external buffer pH to ∼5.5–6 by addition of HCl. After the pH baseline was stable for 2–3 min, the potassium ionophore valinomycin (final concentration 25 nM) was added to the solution. After 5 min, the proton ionophore carbonyl cyanide m-chlorophenylhydrazone, CCCP, (final concentration 0.5 µM) was added to terminate the assay by allowing influx of protons to reach equilibrium. Finally, the solution was back-titrated after 3–5 minutes with HCl. Inhibition of p7-mediated proton transport was monitored with addition of 5 µM rimantadine. The inhibitor was added from concentrated stock solutions in ethanol 5 minutes before initiation of the proton transport assay with HCl. For comparison, a similar assay for influenza A M2 protein (18-60) (protein-to-lipid molar ratio 1∶1000) was also performed. M2(18-60) fragment was pre-solubilized in either methanol or HFIP and results did not change (not shown). For the duration of the experiment, the solution was constantly stirred.

### Liposome flotation assay

In the flotation assay, proteoliposomes were prepared by either the ‘dyalisis’ method after extrusion, or by the ‘addition’ method. Briefly, an appropriate volume of liposome sample containing 50 µg of p7 peptide obtained by either method of incorporation in the modified assay buffer (60 mM NaH_2_PO_4_, 60 mM Na_2_HPO_4_, 85 mM NaCl, pH 7.0), was mixed with sucrose to 20% (w/v) and Na_2_CO_3_ to 0.1 M. This sample was layered at the bottom of a 5-ml centrifuge tube. Successively, 1 ml of 10% (w/v) sucrose solution in assay buffer and 1 ml of assay buffer was layered on top of the sample. Liposome-containing fractions were diluted and centrifuged at 150,000× g for 1 h at 25°C and resuspended in water, or desalted by passing through Econo-Pac® 10 DG column (Bio-Rad), to remove salt and sucrose. Subsequently, the sample was deposited onto a Germanium trapezoidal plate for p7 detection and secondary structure determination. To estimate the quantity of p7 protein in the liposome fraction, a calibration plot was obtained using increasing amounts of p7 (dissolved in HFIP) and dried onto a Ge plate. The amount of p7 was correlated against amide I peak area (1700–1600 cm^−1^) and used to construct a calibration line. For gel-based quantification purposes, dialyzed samples were TCA precipitated, dissolved with NuPAGE sample buffer and electrophoresed as described in the previous section. The PAESC component densities and molecular weights were calculated from egg PA-brain PE-brain PS-egg PC 5∶2∶2∶1, values obtained from Avanti website (www.avantilipids.com). The p7 and sucrose buffer density was calculated using SEDNTERP [30]. For the calculation of PAESC lipid area, the egg yolk PC area was used as a reference [31].

## Results and Discussion

### Purity of synthetic and recombinant p7 protein

**Figure 1:**
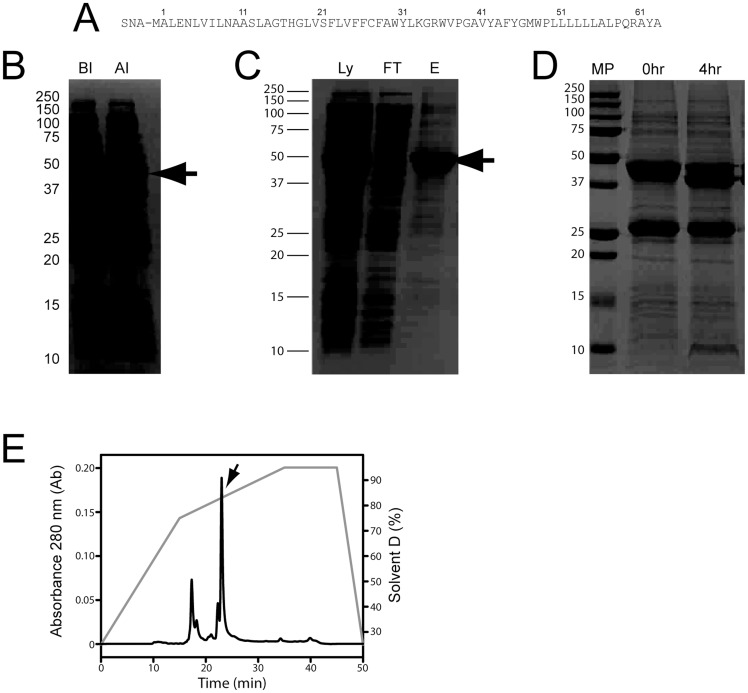
Overexpression and purification of p7 protein from *E. coli*. (A) Amino acid sequence of full-length p7, where extra residues SNAM (see Materials and Methods) are present in the recombinant form and extra M in the synthetic form; (B) A band migrating near 50 kDa was observed after IPTG induction which corresponds to MBP-p7 fusion protein (arrow). BI, before IPTG induction; AI, 16 hours after IPTG induction; (C) Ni^2+^-NTA purification of MBP-p7 with close to 85% purity after elution in 500 mM imidazole. Ly, supernatant of total cell lysate; FT, flow through from Ni^2+^-NTA column; E, eluent from Ni^2+^-NTA column (D) TEV digestion results of MBP-p7 at room temperature, at time 0 and after 4 h incubation with gentle shaking; (E) RP-HPLC purification of p7 with a C3 RP-HPLC chromatography column. The peak corresponding to purified p7 is indicated by an arrow.

Dye release assays have been previously performed with tagged p7 protein, either with FLAG [32], [33] or flu-antigen [25]. In our experiments we have used essentially tag-free p7, either synthetic or recombinant. The sequence of recombinant p7 protein used in the present work is shown in Fig. 1A, which includes 4 extra N-terminal residues (SNAM) [28], whereas the sequence for the synthetic peptide started at Met (residue ‘0’, Fig. 1A).

**Figure 2:**
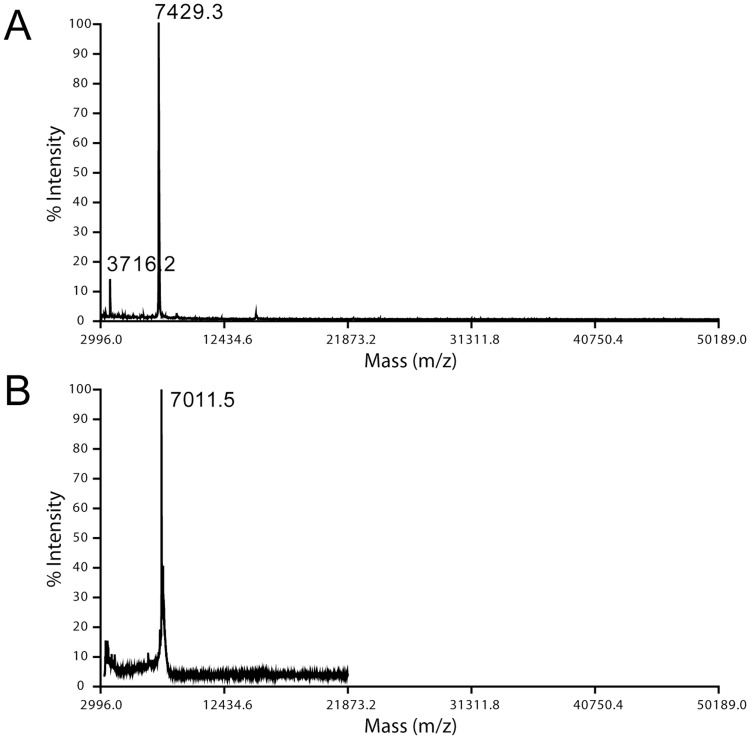
Mass spectrometry of recombinant and synthetic p7 protein. MALDI mass spectra corresponding to the HPLC fraction indicated in Fig. 1E (expected MW 7421.9 Da) (A) and synthetic p7 (expected MW 7017.5 Da) (B). In (A), a double-charged peak also appears at half the expected mass.

The fusion protein 6His-MBP-p7 was expressed at moderate levels after IPTG induction (Fig. 1B). After purification using Ni^2+^-NTA resin (Fig. 1C), the fusion protein was subjected to tobacco-etch virus (TEV) protease cleavage to remove 6His-MBP. Approximately 80% cleavage was achieved in 4 h (Fig. 1D), and no further changes were observed after overnight reaction. The protein was successfully purified by RP-HPLC using an isopropanol gradient (Fig. 1E). Pooled fractions were lyophilized and the purity was assessed by MALDI-TOF MS (Fig. 2A). Synthetic p7 was also purified by RP-HPLC (not shown) and the mass spectrum of the relevant fraction also shows one peak with the expected mass (Fig. 2B). Both synthetic and recombinant p7 were subsequently lyophilized, and further resolubilized in methanol, HFIP, or other solvents, as indicated, prior to reconstitution in lipid bilayers.

### Effect of reconstitution protocol on p7 secondary structure

The secondary structure of p7 was studied by using ATR-FTIR spectroscopy [34] comparing three methods of reconstitution: ‘dialysis’, ‘direct’ and ‘addition’ (see Materials and Methods). In these experiments, freeze-dried p7 was first solubilized in either methanol (M) or HFIP (H), to test the effects of these solvents on p7 conformation.

#### Dialysis method

**Figure 3:**
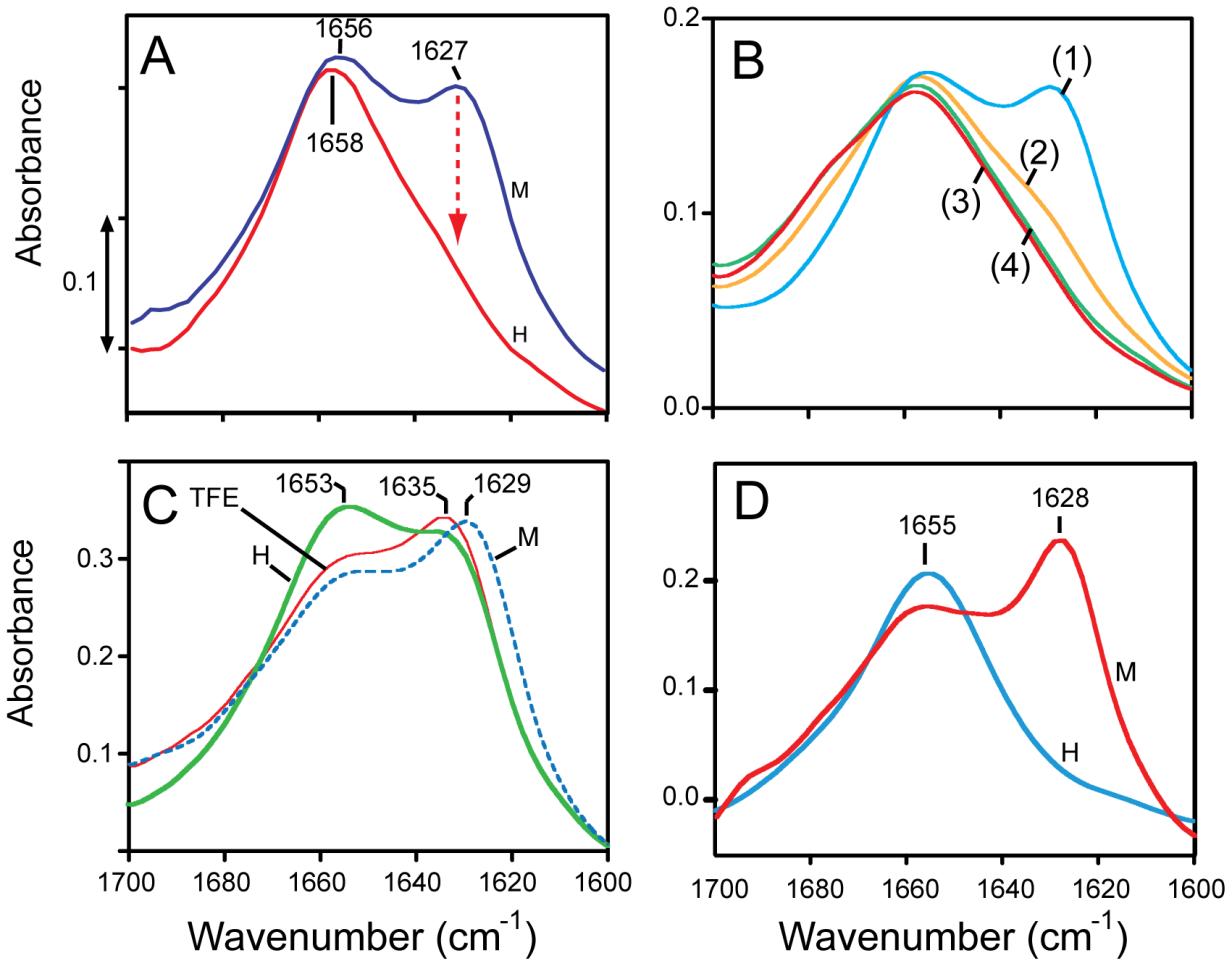
Secondary structure of p7 protein in lipid bilayers. (A) amide I region corresponding to recombinant p7 protein reconstituted into hydrated DMPC bilayers by the dialysis method, after having been solubilized and dried from methanol (M, blue) or HFIP (H, red); (B) Same for a sample reconstituted by the direct method after drying from methanol (1), after freeze-thawing (2), after extrusion (3), and supernatant after 5 min centrifugation of the proteoliposomes at 2,000 g (4); (C) Same for sample obtained by the addition method, where p7 was dissolved at ∼10 mg/ml in the indicated solvents: MeOH, HFIP or TFE; (D), Amide I region of dry recombinant p7 protein obtained after lyophilizing a solution of p7 dissolved in either methanol (M) or HFIP (H).

When recombinant p7 was first dried from HFIP, solubilized in detergent and reconstituted in DMPC lipid bilayers, the amide I spectrum showed that most of the protein is α-helical (trace H in Fig. 3A, henceforth referred to as form A), with a peak centered at 1658 cm^−1^. In contrast, when recombinant p7 was solubilized first in methanol, a band centered at 1627 cm^−1^ appeared prominently, indicating an approximate 60/40% mixture of α-helix and β-strands, respectively (trace M in Fig. 3A, henceforth referred to as form B). Similar differences were observed using a synthetic form of p7 (not shown). Thus, regardless of the sample origin, a dramatic increase in β-structure is observed when the protein was pre-solubilized and dried from a methanol solution. For the α-helical form A, analysis of the amide I region showed that β-structure constitutes approximately 10–15% of the total, whereas the α-helix content was 73%, i.e., ∼48 amino acids. This α-helical content is consistent with the reported 62% obtained for recombinant p7 in DHPC micelles [14], the 70% obtained for synthetic p7 dissolved in 50% TFE [12], and a recent NMR structure of a p7 variant in DPC micelles [20], which is also mostly α-helical and roughly consistent with an α-helical hairpin model with two α-helical TM domains. In contrast, form B obtained after methanol solubilization showed β-structure and α-helical content of 40% and 55%, respectively. This corresponds to ∼37 residues forming α-helices, too short to account for the two predicted α-helical TM domains, but too long for a single TM domain. Therefore, this sample is likely to contain incompletely, or incorrectly, reconstituted p7 protein.

#### Direct method

When we used the direct method of reconstitution, i.e., without a detergent solubilization step, results were identical to the dialysis method. Specifically, samples dried from methanol (M) formed ∼40% β-structure (Fig. 3B, curve 1), whereas those exposed to HFIP (H) were mostly α-helical (data not shown). However, when that sample was subsequently vortexed, freeze-thawed, sonicated and repeatedly extruded, its β-structure content was reduced until the sample became mostly α-helical, i.e., form A (spectra 2–4 in Fig. 3B). This phenomenon was also observed in samples that showed initially a high content of β structure obtained from dyalisis methods (Fig. 3A, see above) or by addition (see below).

#### Addition method

The addition method is used in dye (CF) release assays [24], where a small volume of solvent-solubilized p7 is added to preformed liposomes in aqueous buffer. In this case, p7 formed a significant (∼40%) proportion of β-structure (Fig. 3C, see bands at 1635 and 1629 cm^−1^) regardless of the solvent used for p7 solubilization prior addition to liposomes: methanol, HFIP or TFE.

The presence of β structure in the dyalisis and addition samples when methanol was used (Fig. 3A–B) may be explained by comparison to the spectra of the dry p7 from HFIP or methanol solution (Fig. 3D). In the first case, p7 was almost completely α-helical, whereas in the second case, p7 contained more than 50% of β-structure. However, the CD spectra of p7 in HFIP or methanol solution show no differences, with a high (∼70%) percentage of helical structure (data not shown). Therefore, we conclude that it is the drying procedure than induces β-structure formation, and not the solvent *per se*. This β-structure is retained even after reconstitution in lipids, unless the sample is extruded repeatedly afterwards. This also explains that when added to preformed liposomes, p7 adopts β-structure regardless of the solvent used (Fig. 3C); while p7 is predominantly α-helical in any of these solvents, poor incorporation of p7 in the bilayers after being briefly exposed to the aqueous environment results in β-structure formation.

### Membrane incorporation of p7

Although the samples shown in Fig. 3C show a high percentage of β-structure, it is possible that they are heterogeneous, and that a fraction of the p7 population inserted into membranes is well folded and α-helical. Indeed, the percentage of inserted protein using this ‘addition’ method has been previously reported to range from 10 to 50% using Western blot [24], [32]. Thus, to compare the conformation of the fraction of p7 incorporated to liposome membranes using the dyalisis method - after extrusion -, and that using the addition method, we initially attempted to use a FLAG-p7 and detection by Western-Blot. However, the flagged construct did not elicit CF release using the addition method, even though it was purified in the same way as the non-tagged p7 and that it was pure (not shown). Therefore, further work to test the presence of p7 in the liposomes was performed with the non-tagged p7 protein, and the presence of p7 was directly monitored using SDS-PAGE of the pure protein.

**Figure 4:**
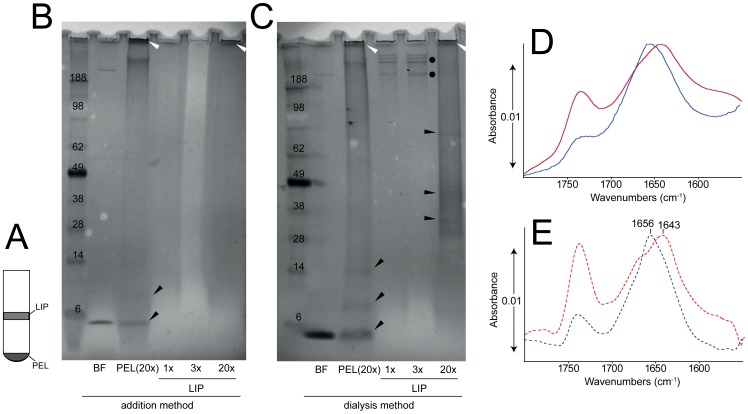
Incorporation of p7 into lipid bilayers. (A) Schematic representation of the flotation system, where fractions with increasing density are found: (LIP), fraction with expected liposome fraction and (PEL), the most dense fraction; (B) SDS-PAGE gel corresponding to samples in the addition experiment: (BF) sample after addition and before liposome flotation, (PEL) sample at the bottom of the tube (see A), (LIP) sample associated to liposomes (see A). Sample loading was nominally 4 µg (1×) except in samples PEL and LIP, as indicated. Arrows and dots are shown for reference to indicate significant bands; (C) same as (B), but for the extruded sample where detergent was removed before extrusion; (D) ATR-IR spectra of the LIP fractions for the ‘addition’ sample (red) and the ‘extrusion’ sample (blue). Spectra for other p7 containing fractions were similar and are not shown; (E) same as (D), after a mild deconvolution (FWHH = 25 cm^−1^ and k = 1.5). The main maxima in the amide I region are indicated.

Thus, two methods, (i) dialysis (extruded) and (ii) addition were used in a flotation experiment to separate membrane-incorporated and non-incorporated p7 protein (Fig. 4A) and to assess its secondary structure. In both samples, p7 was found in both fractions LIP and PEL, and in similar proportion (Fig. 4B–C), i.e., p7 is indeed incorporated in liposome membranes in both samples. A substantial part of the protein appeared as aggregates, accumulated on top of the gel (white arrows) or as clearly defined p7 oligomers (black dots and arrows). The presence of high oligomeric forms may be due to the TCA precipitation, prior to SDS solubilization.

It is noteworthy that a considerable fraction of p7 was also found in the PEL fraction (Fig. 4C, lane PEL(20×)), despite the fact that the bulk extruded sample is α-helical (Fig. 3B). This suggests that the PEL fraction is not merely protein precipitate, but it must also contain lipid. To test this, we analyzed by dynamic light scattering (DLS) the contents of the flotation tube, from top to bottom, in 400 µL fractions. The results showed that liposomes were also present at the bottom of the tube in both samples (Fig. S6 in File S1). The latter suggests that a fraction of the liposomes contains an excessive proportion of p7 protein incorporated, and sinks to the bottom of the tube. The estimated density of the lipid fraction in PAESC liposomes is 1.03 g/mL [35]–[37], whereas the density of the three phases used in the sucrose gradient, 0, 10 and 20%, are 1.01548, 1.05534 and 1.09827 g/mL, respectively. Therefore, pure liposomes should accumulate at the interface between 0 and 10% of sucrose. At the lipid-to-protein ratio molar used in the experiment (100∶1), and assuming uniform incorporation, the density of the liposomes should be 1.0499 g/mL, therefore these liposomes should still accumulate at the 0–10% interface. However, if the incorporation is not homogeneous, e.g., if the lipid to protein ratio is between 70∶1 and 20∶1, the liposomes would be found in the 10% section. The results observed (Fig. S6B in File S1) indicate that some liposomes contain more p7 than that corresponding to a ratio 1∶20, as they are found at the bottom of the 20% sucrose section.

To test if p7 is α-helical when incorporated to membranes, protein isolated in the two conditions from the (LIP) or (PEL) fractions were measured in an ATR-FTIR experiment. Consistent with the results shown in Fig. 3, whereas in the extrusion sample p7 (LIP fraction) adopted a clearly α-helical conformation, the LIP fraction recovered after addition contained more than 50% of β-structure (Fig. 4, D–E). Similar results were obtained in the PEL fraction for both conditions (not shown). Thus, although we cannot discard the possibility that, after addition, some fraction of the population inserted in liposome membranes is in the A form, it is clear that the dyalisis/extruded sample is predominantly α-helical.

### Form A is not responsible for CF release

**Figure 5:**
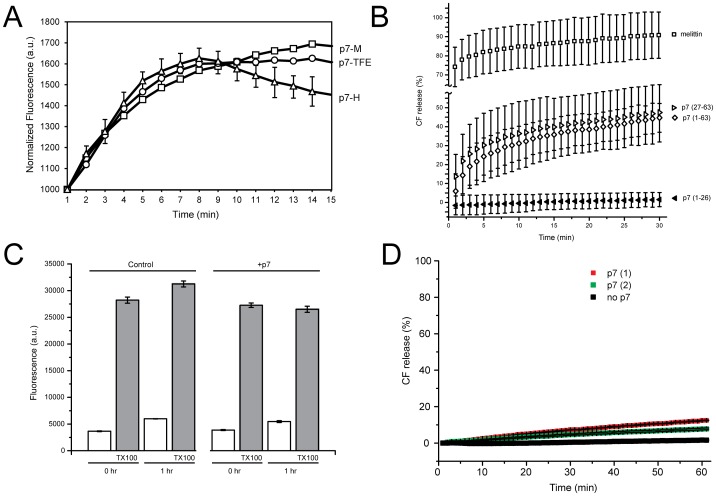
CF release liposome assay. (A) aliquots of p7 in methanol (M), TFE (TFE) or HFIP (H) were added to CF-loaded PAESC liposomes and fluorescence values were measured every minute; (B) Comparison of CF release results of full length p7 versus p7 fragments and melittin. Experimental conditions were as in (A), except that each peptide was added in methanol and peptide∶lipid molar ratio was 1∶10. Error bars (1 SD) are the result of 3 independent measurements; (C) same experiment, but p7 was incorporated into the liposomes by the dyalisis method after freeze-thaw and extrusion. Fluorescence values were measured immediately (0 h) after removing excess CF and after one hour (1 h). Liposome without p7 was used as control, and TX100 was added to measure total amount of CF incorporated. Error bars (1 SD) are shown only for one trace for clarity, and are the result of 3 independent measurements; In (A) and (B), no effect was observed when the liposomes were preincubated with up to 100 µM rimantadine; (D) kinetics of CF release when liposomes were extruded, where p7(1) and p7(2) represent samples containing p7 reconstituted with or without detergent solubilization steps, respectively.

p7 has been shown previously to permeabilize liposomes and induce release of the large dye carboxyfluorescein (CF) [7]. We observed no significant differences in the magnitude or in the kinetics of CF release when p7 solubilized in TFE, HFIP or methanol was added to PAESC liposomes (Fig. 5A). This is consistent with the similar β-structure content of these preparations (Fig. 3C).

To test if an incorrectly incorporated form of p7 is responsible for this CF release, we compared CF-release results from ‘addition’ experiments using p7(1-63) with its fragments, p7(27-63) and p7(1-26), that cannot form an α-helical hairpin or a native p7 structure. p7(27-63) corresponds to TM2 (more hydrophobic) and the extramembrane loop, whereas p7(1-26) corresponds to TM1 (more hydrophylic).

The more hydrophobic fragment p7(27-63) was as efficient as full length p7 in releasing CF, although less than melittin (Fig. 5B), whereas p7(1-26) did not elicit CF release. For comparison, we used a similar hydrophobic protein with a single transmembrane (TM) domain, the peptide M2 (18-60) from influenza A, which caused only a minor CF release (Fig. S7 in File S1), suggesting that CF release may be specific for the C-terminal region of p7. In summary, these results show that correct incorporation and native conformation of full length p7 is not necessary to elicit CF release, and that insertion of the TM2 fraction of p7 into lipid bilayers may be sufficient, based on the more hydrophobic nature of TM2. We note that in a recently published structural model of p7 [20], TM1 and TM2 would correspond to three helical segments H1–H3, i.e., H1 and first half of H2 (TM1), and second half of H2 and H3 (TM2). In that model, p7(27-63) is not the pore-lining sequence and forms the ‘lipid facing’ part of the molecule.

To test if form A is responsible for CF release, we incorporated p7 into CF-containing PAESC liposomes by the dyalisis method, freeze-thawing and extrusion, which should result in p7 incorporation in membranes in an α-helical form A (see Fig. 3B and Fig. 4). However, after removing non incorporated CF, no significant differences in fluorescence were observed between the CF-loaded control (without p7) and sample (with p7) liposomes (Fig. 5C, white bars). Differences were not observed even 1 h after removing non-incorporated CF. Most or all CF was still encapsulated, as shown by the large increase in fluorescence after Triton X-100 addition in both preparations (Fig. 5C, grey bars), and kinetics of CF release were also similar to the addition of only methanol to the liposomes (Fig. 5D). Thus, the result of this experiment is incompatible with form A being responsible for CF release.

### Localization of p7 permeabilizing domain

CF release is affected by mutations that change p7 conformation or liposome insertion, e.g., Ala mutants in a genotype 1b J4 context K33A/R35A, H17A, G39A and P49A) [32]. These authors showed that most of these mutants could form oligomers in detergent, and could insert in membranes. However, K33A/R35A was the least able to produce CF release, and also was the least able to insert in liposomes in a Ficoll gradient experiment. The basic residues K33 and R35 have been located in a loop region separating TM1 and TM2 in previous reports [12], [14]. These residues also appear between two helical segments in a recent structure of p7 in DPC micelles [20], where TM1 is proposed to line the lumen of the channel.

The region encompassing these residues in the interhelical loop region were predicted to be α-helical in 50% TFE, but were not α-helical in DHPC micelles [12], [14] (Fig. S8 in File S1). In another study [38], a synthetic peptide corresponding to that loop, p7(F25-Y42), produced only β-structure in presence of lipid bilayers, and this stretch of amino acids was found to be the most efficient at disrupting liposome integrity. Regions in and around the loop contain abundant β-branched side chains (V, I), and bulky residues (Y, F,W), which disfavor α-helical conformation [39]–[41]. Thus, this small loop region is a candidate to elicit CF release, although alone does not account for the extent of β-structure found in form B (Fig. 3), which may be likely contributed by either TM1 or TM2.

To identify which regions form β-structure in form B, we attempted to perform native chemical ligation between a C-terminal thioester p7(1-26), encompassing TM1, and p7(27-63), encompassing TM2, with an N-terminal cysteine [42] Cys27 (Figs. S2 and S3 in File S1). A successful ligation with differentially ^13^C labeled fragments (roughly corresponding to TM1 and TM2 [12], [14]) would produce a hybrid sample the results of which could be easily interpreted by IR. The ligation was successful, but did not produce sufficient yield (Figs. S4 and S5 in File S1). Nevertheless, these two peptides were analyzed independently by ATR-FTIR.

**Figure 6:**
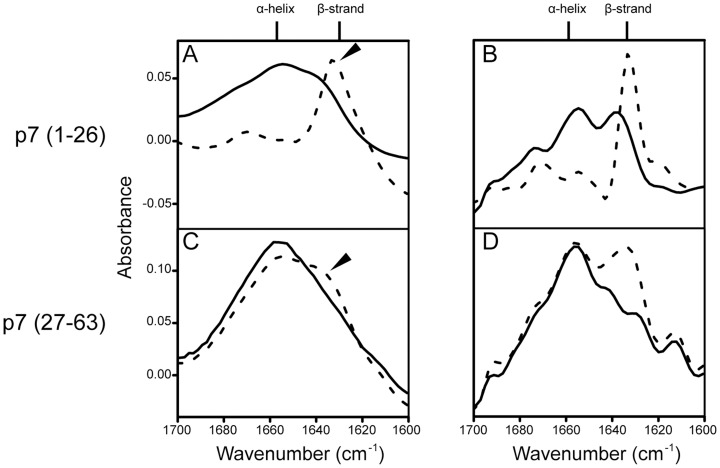
Secondary structure of N- and C-terminal fragments of p7. Infrared amide I region of (A) fragment p7(1-26), and its Fourier self deconvolved spectrum (B); C and D, same for fragment p7(27-63). Samples pre-solubilized and dried from methanol and HFIP are indicated as dotted and solid lines, respectively.

The synthetic N-terminal peptide p7(1-26) was totally insoluble in TFE or in detergent. The difficulty in handling and purification of the N-terminal fragment of p7 has been reported previously for p7(1-34) [12]. After solubilization in methanol and ‘direct’ reconstitution in DMPC liposomes, p7(1-26) produced a spectrum in the amide I consistent with ∼100% β-structure (Fig. 6, A–B, dotted line). A high content (∼50%) in β-structure was also observed when the peptide was previously solubilized in HFIP (Fig. 6, A–B, solid line). The C-terminal peptide, p7(27-63) in contrast, was more α-helical in both conditions tested (Fig. 6, C–D), although with more β-structure in the methanol condition. Nevertheless, it is remarkable that neither TM domains of p7, especially TM1, behaves like typical α-helical TM domains in similar membrane proteins. For example, α-helical TM domains in integrins [43], phospholamban [44], influenza A M2 [45], SARS-CoV E [29], or RSV SH [46] produce sharp amide I bands in the infrared spectrum consistent with ∼100% α-helix.

In agreement with the above, it has been noted that the N-terminal half of p7 is more hydrophilic [15], and the predicted hydrophobic segment (starting at residue ∼13) is too short to form a TM α-helix. In contrast, TM2 has the appropriate length and hydrophobicity to constitute a ‘real’ α-helical TM. Based on the above considerations, TM1 may be more difficult to incorporate in lipid bilayers than TM2, and more likely to fold forming β-structure, presenting itself as a totally or partially exposed peptide to the aqueous solution under certain reconstitution conditions.

### Proton channel activity of p7 form A

Having examined the conditions in which we can obtain form A, consistent with an α-helical hairpin, or a mostly helical structure [12], [20], we tested the ability of form A in transporting protons. Proton channel activity of p7 has been observed in infected mammalian cells, acting to prevent acidification of intracellular vesicles [8], and was found to be essential for productive HCV infection. However, results from an *in vitro* assay using purified protein have not been reported to our knowledge.

**Figure 7:**
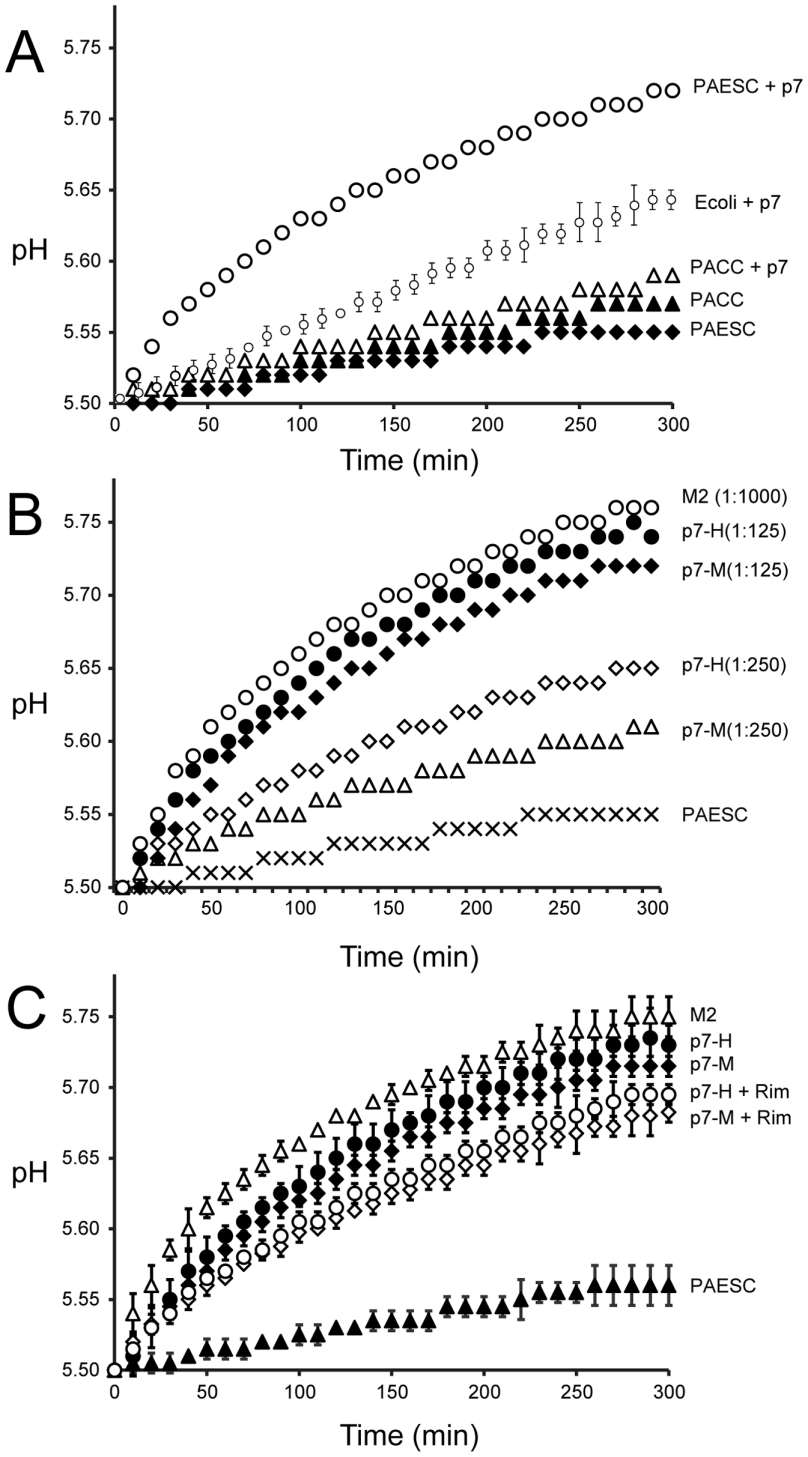
Liposome based proton transport assay. (A) Proton transport mediated by recombinant p7 pre-solubilized in HFIP at the p7/lipid 1∶125 molar ratio. Three different lipid mixtures were used for the assay, *E. coli* lipids, PA/PE/PS/PC (PAESC), 5∶2∶2∶1 (w/w) and PA/PE/PC/Cholesterol (PACC), 5∶2∶2∶1 (w/w). PACC and PAESC represent negative control, i.e., liposomes without p7; (B) Proton transport mediated by recombinant p7, where p7 was solubilized in HFIP (H) or methanol (M) and mixed with PAESC lipids at protein to lipid ratios indicated. For comparison, a trace using M2(18-60) in *E. coli* lipids in also shown at the protein to lipid molar ratio indicated; (C) Same as above, showing the effect of 5 µM rimantadine (+Rim). PAESC, negative control, i.e., liposome without p7.

We performed a proton transport assay where the alkalinization of the external solution is measured after generation of a pH gradient and addition of valinomycin (see Materials and Methods). First we tested if p7 dried from HFIP (p7_H_) or methanol (p7_M_) and reconstituted by the direct method after several extrusion cycles – where form A will be obtained (Fig. 3B, curve 4) - would be equally able to transport protons. After trying several lipid compositions, p7_H_ (Fig. 7A) was found to be more active in the mixture PAESC (PA/PE/PS/PC 5∶2∶2∶1 w/w), and similar results were obtained for p7_M_. The proton channel activity was less efficient in *E. coli* lipids, and completely inactive in the mixture PACC (PA/PE/PC/Chol 50∶20∶20∶10 w/w) (other lipid compositions tested are not shown). The PAESC composition was also previously found to be more suitable for storage and stability of liposomes in CF release high-throughput assays [26]. The pH changes observed were significantly larger than those measured with control liposomes (trace PAESC in Fig. 7A, ΔpH_5 min_ = 0.03±0.01).

The total pH change 5 min after valinomycin addition (Fig. 7B) was significantly larger (one-way ANOVA, P<0.05) for p7_H_ (ΔpH_5 min_ = 0.22±0.03) than for p7_M_ (ΔpH_5 min_ = 0.20±0.01), probably due to p7_M_ incomplete conversion to form A after extrusion. Nevertheless, both preparations were able to transport protons in a concentration dependent fashion. For comparison, we used influenza A M2 fragment (18-60), a ‘classical’ proton channel, which was more efficient than p7 (ΔpH_5 min_ = 0.25±0.03), even at a protein to lipid ratio ∼9 times lower (see Materials and Methods). Rimantadine (5 µM) partially inhibited the proton channel activity of p7 (Fig. 7C), consistent with previous observations [8]. Overall, these results show that form A, consistent with an α-helical hairpin, is responsible for proton transport. We could not test if form B can also transport protons because the proton transport assay requires extrusion to produce unilamellar liposomes, and this inevitably converts form B to A (Fig. 3B).

**Figure 8:**
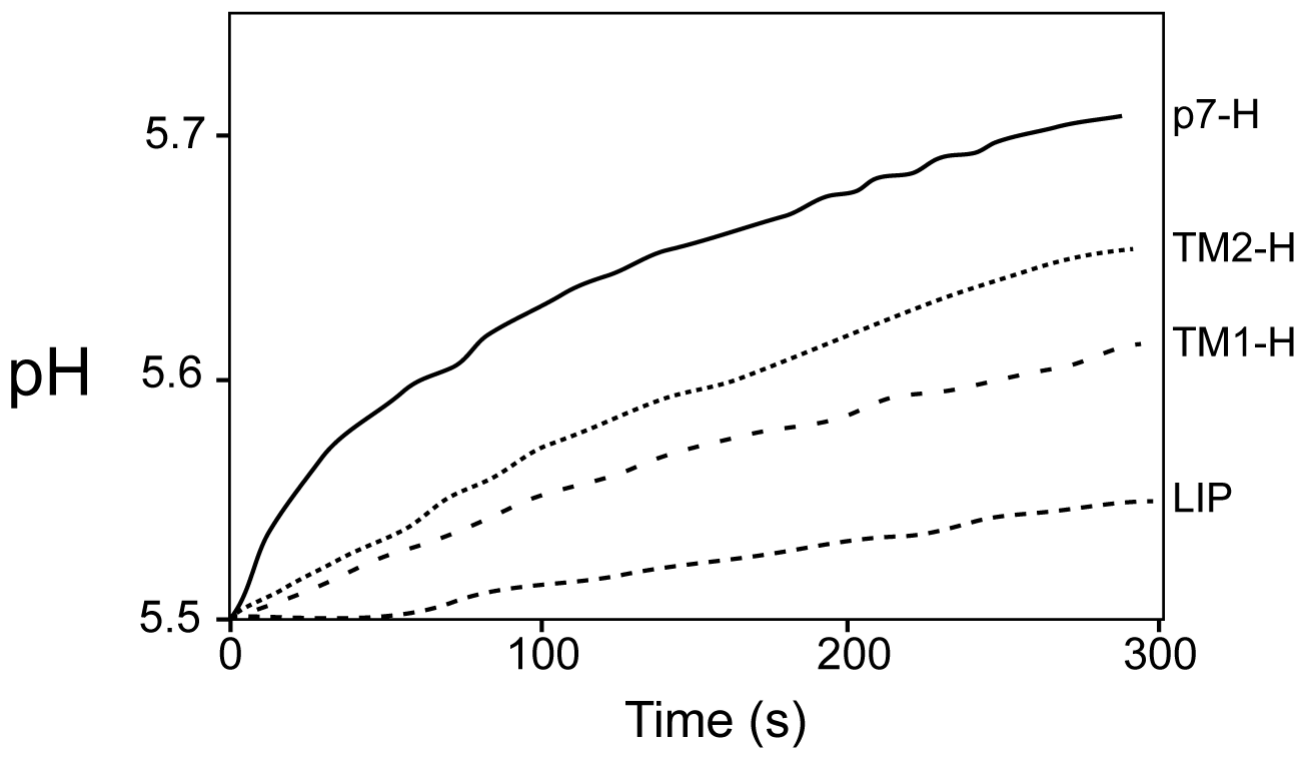
Proton transport of p7 and its fragments. Comparison of liposomal proton transport mediated by p7 full length, p7-TM1 fragment and p7-TM2 fragment solubilized in HFIP prior mixing with lipids (protein to lipid molar ratio 1∶125); LIP, negative control without peptide.

Then we tested if fragments encompassing TM1 and TM2 can transport protons (Fig. 8). Both fragments were able to transport protons to some extent, although the linear plot suggests that this permeability may not be specific. The total pH change 5 min after valinomycin addition was still significantly larger (one-way ANOVA, P<0.05) for TM2 p7(27-63) (ΔpH_5 min_ = 0.15±0.01) than for TM1 p7(1-26) (ΔpH_5 min_ = 0.1±0.01). However, this may simply reflect a more difficult incorporation of TM1 in lipid bilayers.

### Model for A and B forms of p7

**Figure 9:**
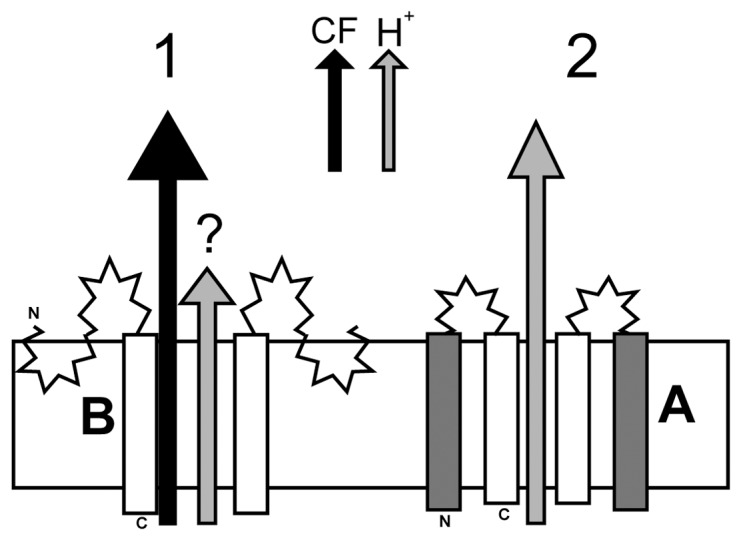
Schematic view of the proposed two forms of p7 (A and B). (1) In form B, TM2 is embedded in the lipid bilayer and the extra β-structure is contributed by exposure of TM1 to the aqueous environment. This form is able to release CF (black arrow), but not protons (grey arrow). As part of the B form extends into the extramembrane domain, it is likely to participate in fusion events or membrane destabilization; (2) Form A has two TM domains, TM1 and TM2, separated by a loop, where TM2 lines the lumen of the channel. This form is unable to release CF, but it is able to transport protons.

The conclusion of these experiments are summarized in a cartoon representation (Fig. 9), where form B has only one TM inserted in the lipid bilayer (TM2), whereas interhelical loop and TM1 form predominantly β-structure that may be partially inserted in the membrane. We propose that this form is responsible for CF release (see black arrow). Form A, in contrast, is not able to allow CF release, although it is able to transport protons (grey arrow).

These models assume that TM2 is the site for inhibition. This is supported by the observed direct interaction between the inhibitor amantadine and TM2 leucines 51–57, which have been shown unequivocally to be part of a membrane inserted α-helix [20], [47], and Leu to Ala mutations at this region of TM2 produced an amantadine resistant p7 mutant [32]. However, amantadine and rimantadine have also been reported to interact with some residues in TM1 when forming an α-helical hairpin [20], [32], [47]. Thus we propose that these drugs interact with membrane-inserted TM2, possibly increasing its rigidity (and preventing CF release) when form B is present, and interact with both TM1 and TM2 (inhibiting proton channel activity) when form A is present. Indeed, that amantadine is able to interact with both TM1 and TM2 has been recently confirmed experimentally using p7 in DPC micelles, although the strain used is quite different from ours [20]. The p7 sequence (subtype 5a, strain EUH1480) in that report is less than 50% identical to the sequence used here (1a, H77). Overall, our results suggest that, although a CF release assay may be able to detect p7 channel inhibitors because of the proposed dependency of CF release on membrane-inserted TM2, a proton or ion transport assay is more relevant to discover channel blockers.

From the model shown in Fig. 9, once could argue that form B cannot transport protons or ions because TM2 is not lining the chanel in p7. However, although a recent NMR based model places TM1 as lining the lumen [20], this is still a controversial point. Support for this arrangement is found in the physico-chemical properties of TM1 and molecular dynamics simulations [16], [19], and the observed partial inhibition of p7 channel activity by Cu^2+^, but not by Mg^2+^ [17]. Peptides corresponding to TM2 or TM1, e.g., p7(35-63) or p7(1-34) easily aggregate, so that individual channel activity of TM1 or TM2 cannot be properly measured [12]. In the latter paper, peptide TM1 p7(1-34) showed some channel activity that was probably non-specific, because inhibition data was not reported, and the peptide had not been purified [12]. Other reports were unable to measure channel activity for this peptide [5], and we show that purified peptide p7(1-26) is not even α-helical in presence of lipid bilayers after methanol exposure, and it is only partially helical after HFIP exposure.

### Possible relevance of forms A and B *in vivo*

Although we propose that form B is simply an artifact, it is possible that when the p7 polypeptide is cleaved during processing, an equilibrium is established between form A and B, where TM1 is totally or partially exposed to the aqueous domain, leading to a coexistence between these two conformations, and this may explain different topologies reported in the literature. This is not rare in viral proteins; the N terminus of HCV NS4B has been reported to assume dual TM topology, with possible distinct functions, on each side of the ER membrane [48]. In addition, the hepatitis B virus surface L-glycoprotein also adopts different TM topologies, acting during virus assembly as a matrix-like protein, and in virus entry as a receptor-binding protein [49]. Lastly, the Newcastle disease virus fusion protein exists in two topological forms with respect to membranes, one of which has been proposed to be involved in fusion [50].

Indeed, some functions of p7 are independent from its ion or proton channel activity because they are not affected by either channel inactivating mutations, or treatment with rimantadine [8]. For example, p7-mediated localization of NS2 protein to sites of viral replication in lipid raft regions [9], or the transfer of HCV core protein from lipid droplets to the ER, which depends on interaction between p7 and NS2 [10]. Incidentally, in this role, the interaction between p7 TM2 and NS2 TM1 was proposed as specially important. p7 also interacts with other viral structural and non-structural proteins that are important to promote virus assembly and release [2], [51]–[53]. Lastly, although the present results have been obtained with p7 from subtype 1a, it is likely that these results can be extrapolated to other studies that used different subtypes of HCV p7 because of the >80% identity between sequences of subtypes 1a, 1b and 2a.

Viruses are economical in their use of resources and it is often encountered that a single protein performs a variety of tasks, being involved in invasion of the host cell to evasion from the host defense system, and conformational flexibility has been recognized as one of the features necessary to provide these functionalities [54].

## Supporting Information

File S1 **This file contains:** **Figure S1.** Single channel recordings of HCV-p7 (synthetic) ion channel in DPhPC lipid membranes. (A) HCV-p7 was incorporated by adding a 10 µM methanol solution of the protein onto a previously formed lipid membrane seal; (B) HCV-p7 ion channel activity is blocked by HMA in a full trace of different voltages after five minutes. Similar results were obtained for recombinant p7 (not shown). **Figure S2.** Gel electrophoresis of p7 (recombinant, Rp7, and synthetic, p7) and its synthetic fragments (1-26) and (27-63). Left: 15% Tris-glycine gel in reducing conditions; Right: NuPAGE gel under non-reducing conditions (see Materials and Methods for details). **Figure S3.** Mass spectrometry of synthetic fragments 1-26 thioester, with expected mass of 2800 Da (A) and 27-63 with expected mass 4,044 Da (B). **Figure S4.** (a) Sequence of HCV p7 protein (top) with ligation point (Cys, downward arrow); (b) HPLC elution profile of HCV p7 protein ligation mixture, at 0 h (elution of only fragments A and B) and at 36 h (A fragment consumed); (c) SDS page of p7 ligation product after 5 h of reaction (arrow), and peptides A and B (star). The p7 product remained inside the column and was eluted with isopropanol (see Fig. S5 in File S1). **Figure S5.** (a) MALDI mass spectra of HCV p7 protein ligation mixture after 36 h and (b) ligated product p7 purified by HPLC. **Figure S6. Average count rate versus sample fraction in flotation experiment.** (**A**) extruded sample where detergent was removed before extrusion; (B) sample after addition of p7; (C) Autocorrelation function (ACF) for the samples in (A) and (B), for each of the fractions indicated on the left. The sucrose concentration is shown on the right. The calculated size of the last fraction (#13) in samples extruded and addition was 400 and 320 nm, respectively. For the samples in 10% sucrose (fractions #4 to #7), the average size was smaller, 120–150 nm in both samples (not shown). **Figure S7. CF release kinetics following addition or extrusion.** (A) Comparison of CF release in the addition method using melittin, p7, M2 (18-60) and only methanol. Details are indicated in the Materials and Methods section of the manuscript. **Figure S8.** Regions of p7 that adopt α-helical conformation according to previous reports. The stretches corresponding to α-helical regions (underlined) reported for p7 in 50% TFE [12] (A) and DHPC micelles [13] (B), according to NMR experiments. Both sequences correspond to genotype 1b but to different strains, HCV-J (A) and J4 (B). The approximate regions that span TM1 and TM2, and the loop region that produced maximal membrane perturbation [38] (grey bar) are indicated. Differences in the sequences are indicated by a bold letter.
(DOCX)
