# Supplementary material for: Two Different Conformations in Hepatitis C Virus p7 Protein Account for Proton Transport and Dye Release
Source: PLoS One. 2014 Jan 7;9(1):e78494. doi: 10.1371/journal.pone.0078494 (PMC3883635; doi:10.1371/journal.pone.0078494)
Supplement: File S1 — This file contains: Figure S1. Single channel recordings of HCV-p7 (synthetic) ion channel in DPhPC lipid membranes. (A) HCV-p7 was incorporated by adding a 10 µM methanol solution of the protein onto a previously formed lipid membrane seal; (B) HCV-p7 ion channel activity is blocked by HMA in a full trace of different voltages after five minutes. Similar results were obtained for recombinant p7 (not shown). Figure S2. Gel electrophoresis of p7 (recombinant, Rp7, and synthetic, p7) and its synthetic fragments (1-26) and (27-63). Left: 15% Tris-glycine gel in reducing conditions; Right: NuPAGE gel under non-reducing conditions (see Materials and Methods for details). Figure S3. Mass spectrometry of synthetic fragments 1-26 thioester, with expected mass of 2800 Da (A) and 27-63 with expected mass 4,044 Da (B). Figure S4. (a) Sequence of HCV p7 protein (top) with ligation point (Cys, downward arrow); (b) HPLC elution profile of HCV p7 protein ligation mixture, at 0 h (elution of only fragments A and B) and at 36 h (A fragment consumed); (c) SDS page of p7 ligation product after 5 h of reaction (arrow), and peptides A and B (star). The p7 product remained inside the column and was eluted with isopropanol (see Fig. S5 in File S1). Figure S5. (a) MALDI mass spectra of HCV p7 protein ligation mixture after 36 h and (b) ligated product p7 purified by HPLC. Figure S6. Average count rate versus sample fraction in flotation experiment. (A) extruded sample where detergent was removed before extrusion; (B) sample after addition of p7; (C) Autocorrelation function (ACF) for the samples in (A) and (B), for each of the fractions indicated on the left. The sucrose concentration is shown on the right. The calculated size of the last fraction (#13) in samples extruded and addition was 400 and 320 nm, respectively. For the samples in 10% sucrose (fractions #4 to #7), the average size was smaller, 120–150 nm in both samples (not shown). Figure S7. CF release kinetics following addition [file pone.0078494.s001.docx]

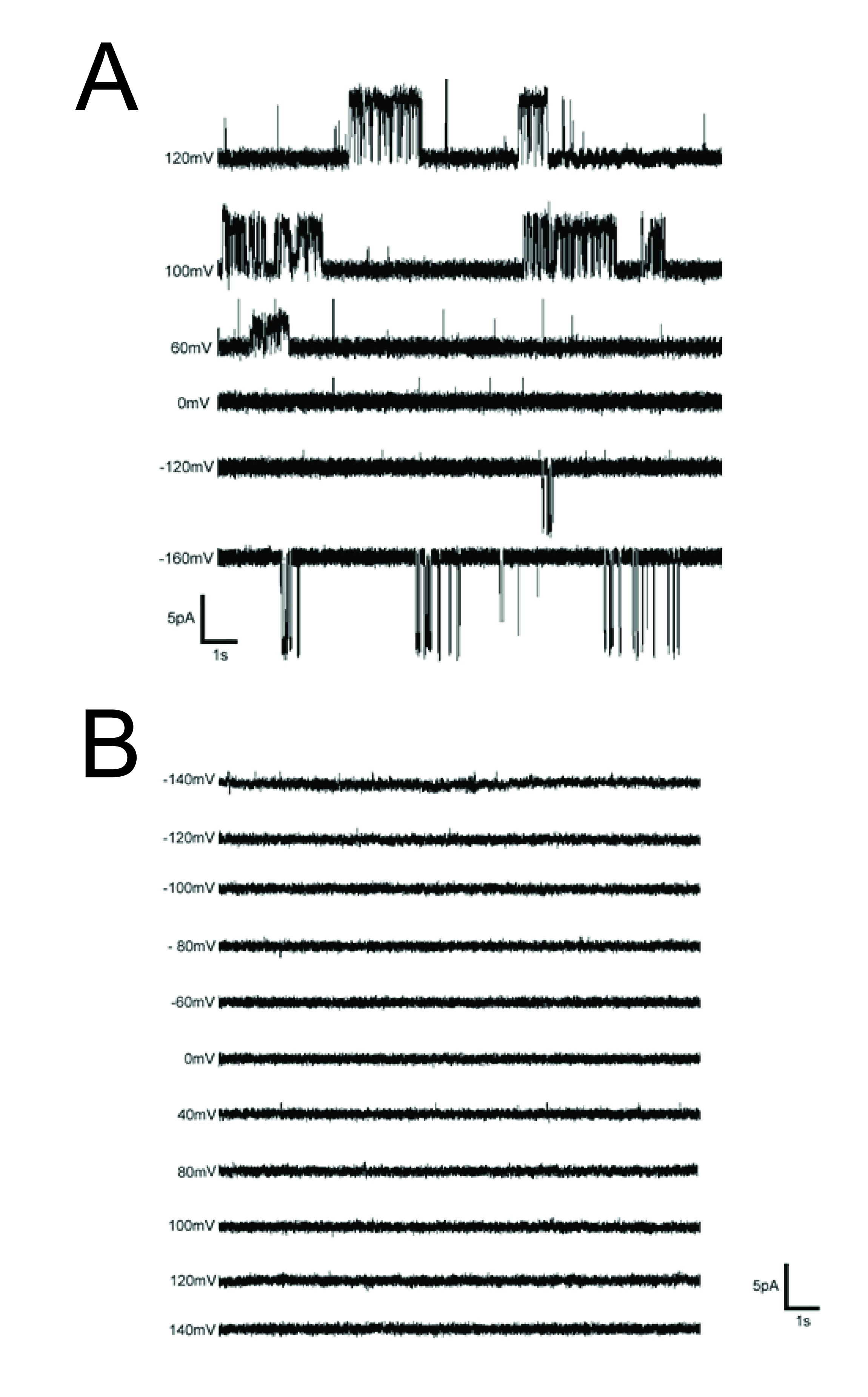


**Figure S1.** Single channel recordings of HCV-p7 (synthetic) ion channel in DPhPC lipid membranes. (A) HCV-p7 was incorporated by adding a 10 μM methanol solution of the protein onto a previously formed lipid membrane seal; (B) HCV-p7 ion channel activity is blocked by HMA (100 μM) in a full trace of different voltages after five minutes. Similar results were obtained for recombinant p7 (not shown).


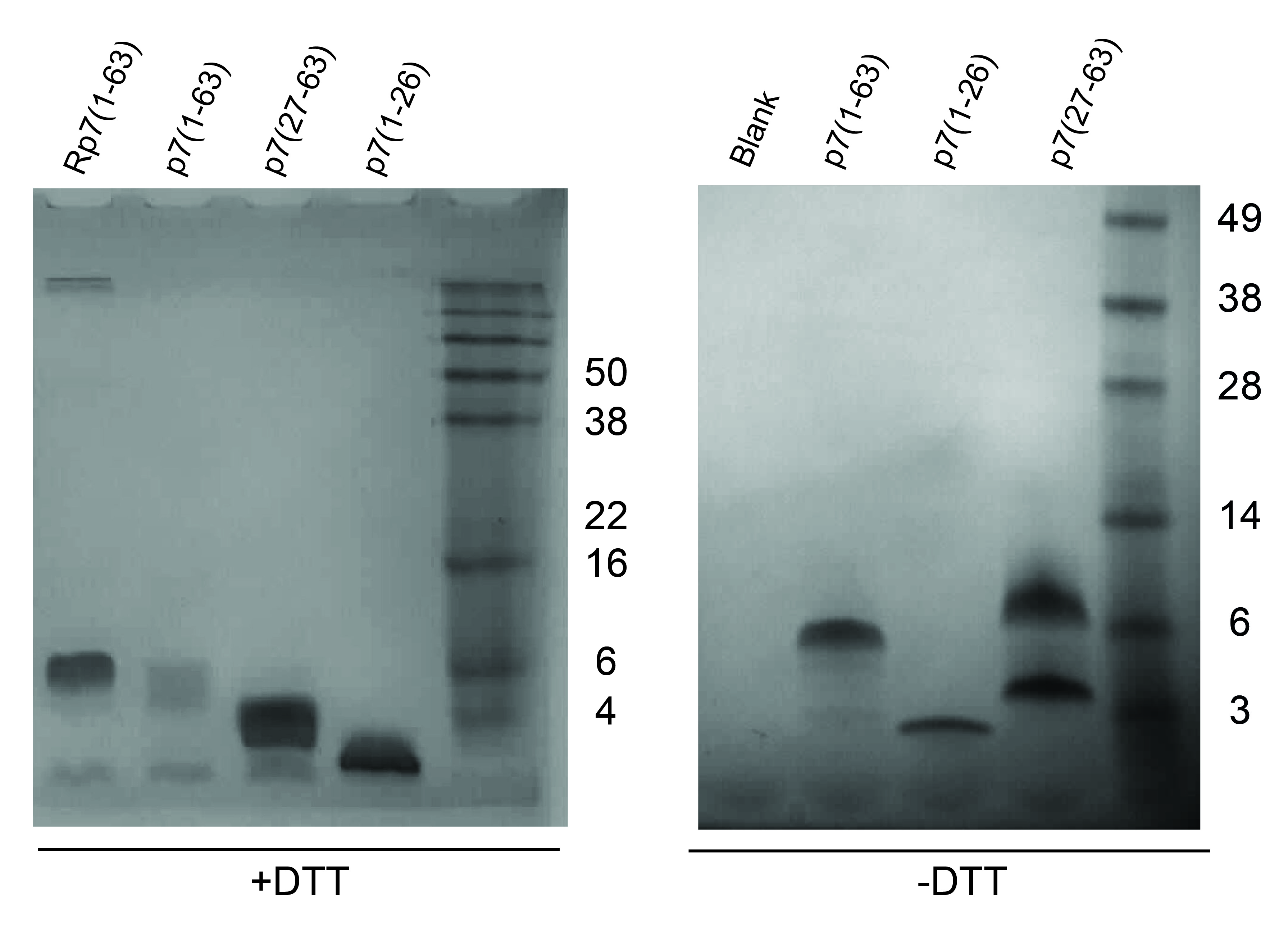


Figure S2. Gel electrophoresis of p7 (recombinant, Rp7, and synthetic, p7) and its synthetic fragments (1-26) and (27-63). Left: 15% Tris-glycine gel in reducing conditions; Right: NuPAGE gel under non-reducing conditions (see Materials and Methods for details).


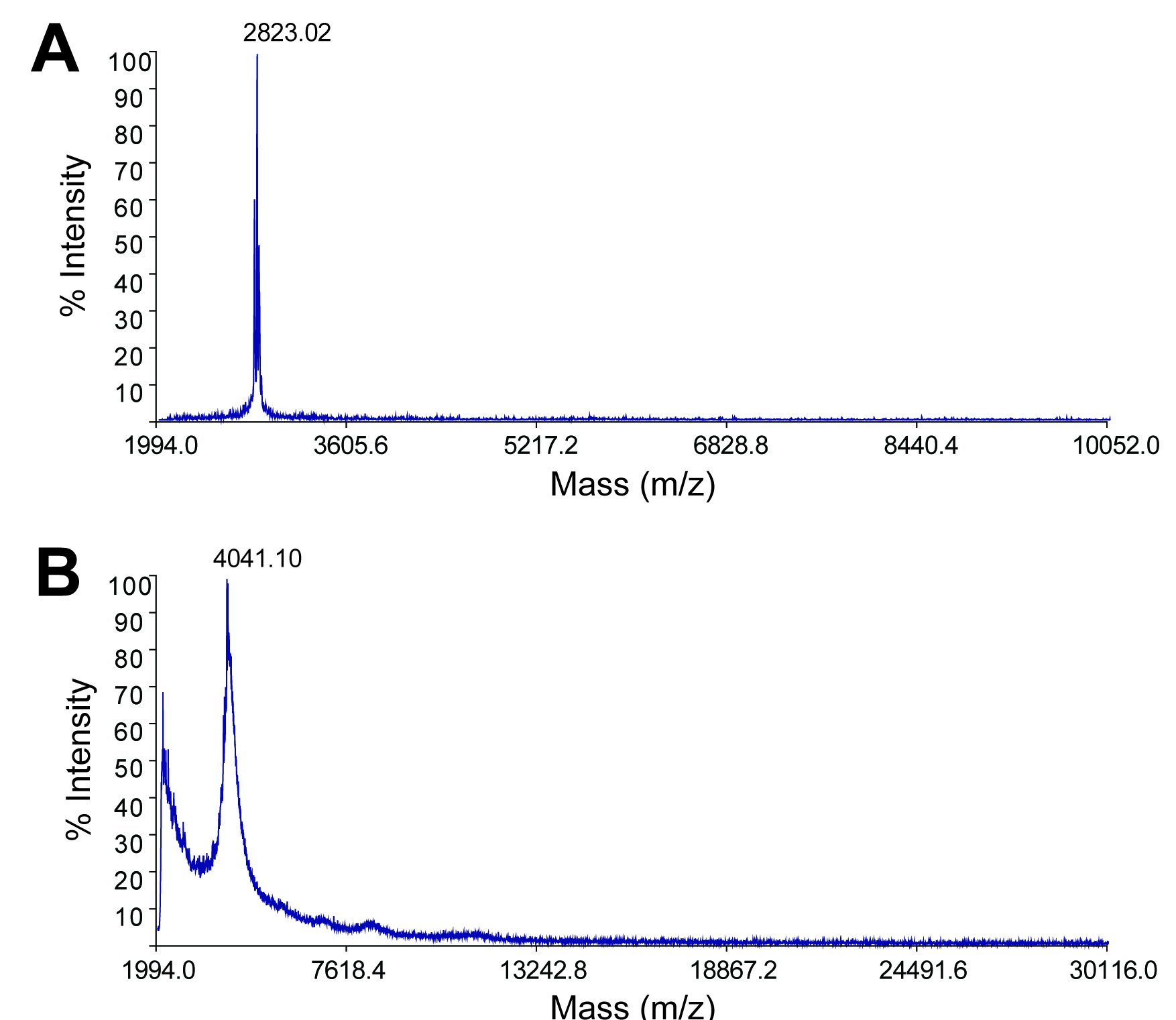


**Figure S3.** Mass spectrometry of synthetic fragments 1-26 thioester, with expected mass of 2800 Da (A) and 27-63, with expected mass 4,044 Da (B).

**
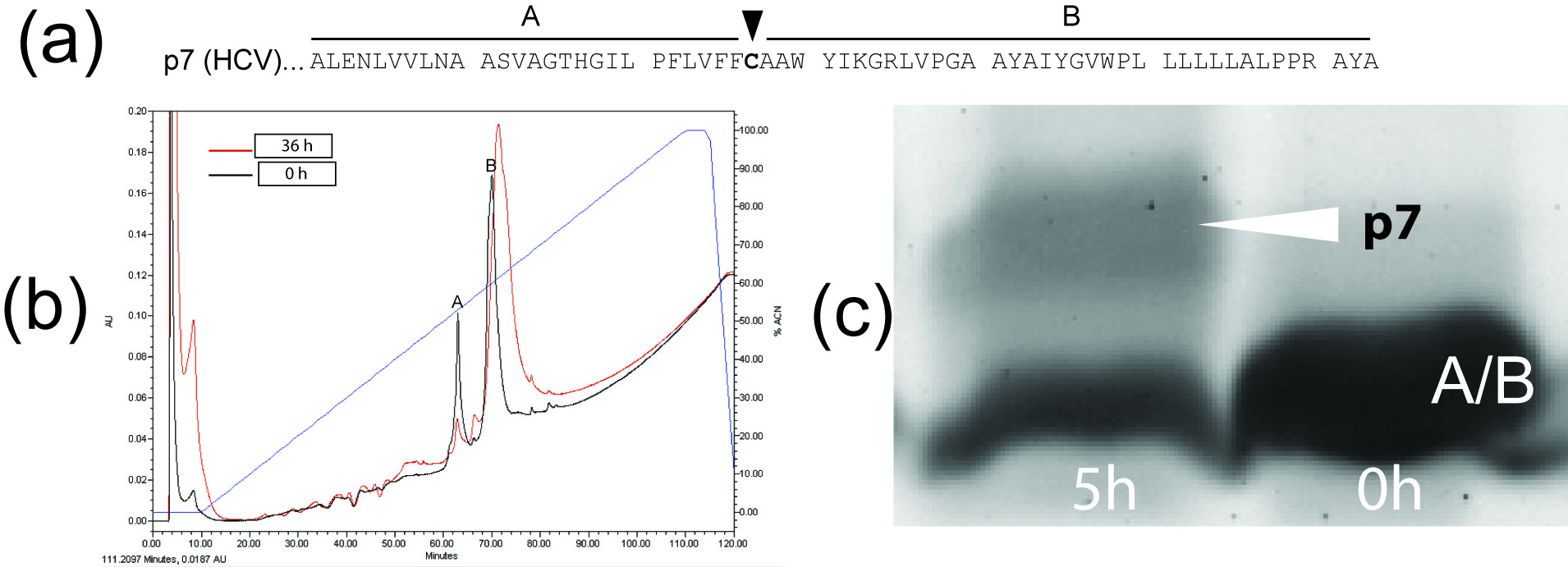
**

**Figure S4.** (a) Sequence of HCV p7 protein (top) with ligation point (Cys, downward arrow); (b) HPLC elution profile of HCV p7 protein ligation mixture, at 0h (elution of only fragments A and B) and at 36 h (A fragment consumed); (c) SDS page of p7 ligation product after 5 h of reaction (arrow), and peptides A and B (star). The p7 product remained inside the column and was eluted with isopropanol (see Fig. S5).

**
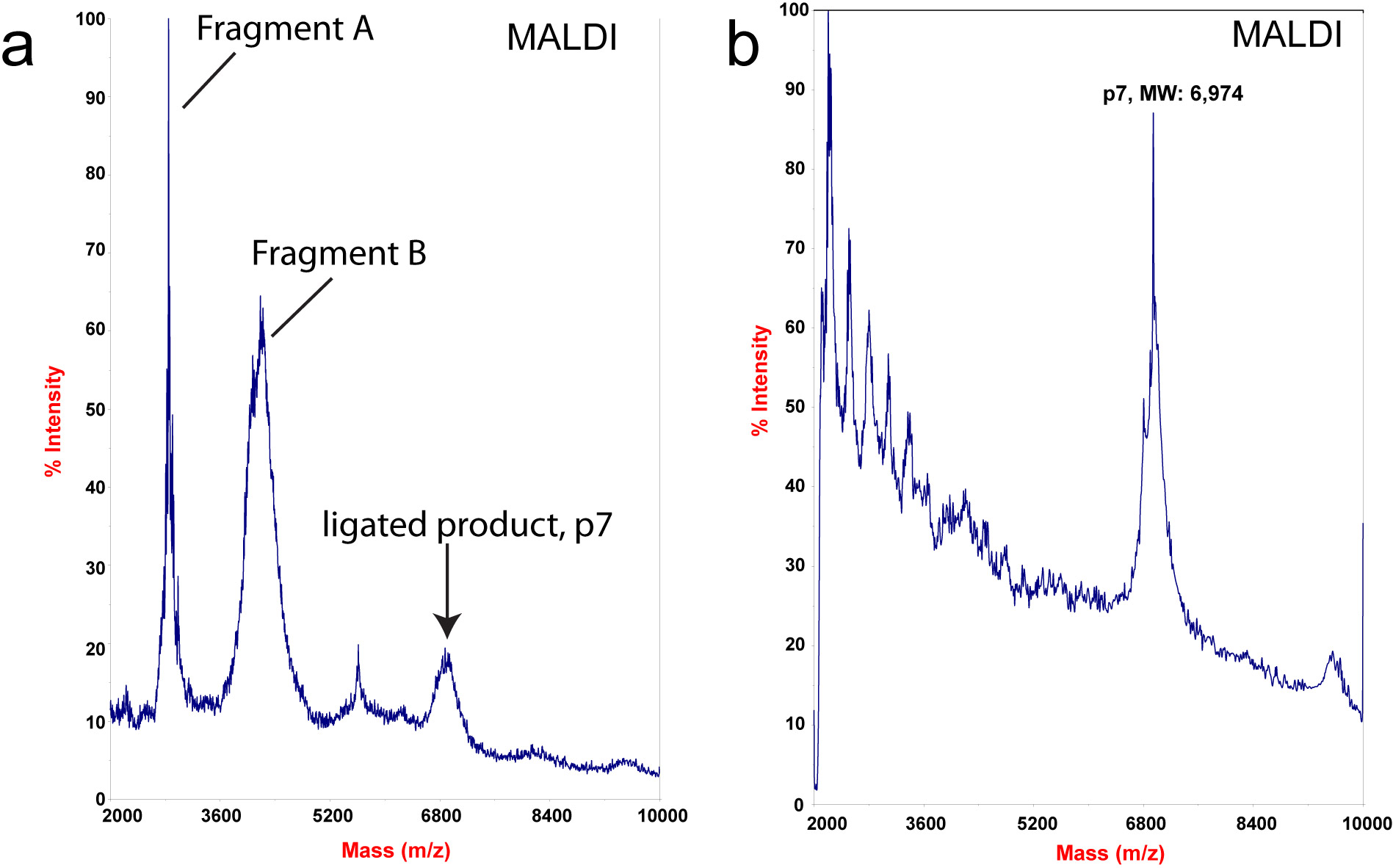
**

**Figure S5.** (a) MALDI mass spectra of HCV p7 protein ligation mixture after 36h and (b) ligated product p7 purified by HPLC.


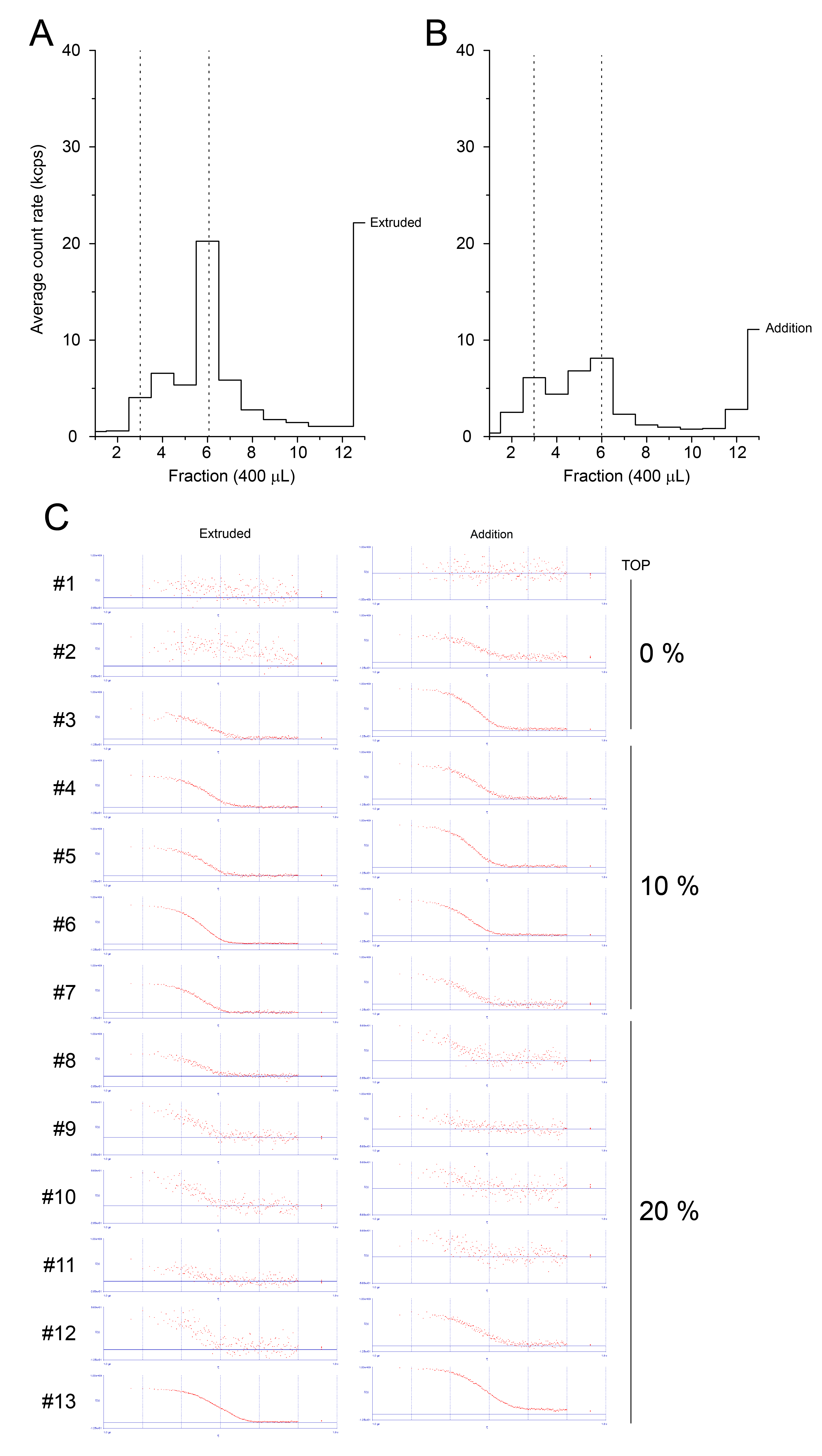


**Figure S6. Average count rate versus sample fraction in flotation experiment. (A)** extruded sample where detergent was removed before extrusion; (B) sample after addition of p7; (C) Autocorrelation function (ACF) for the samples in (A) and (B), for each of the fractions indicated on the left. The sucrose concentration is shown on the right. The calculated size of the last fraction (#13) in samples extruded and addition was 400 and 320 nm, respectively. For the samples in 10% sucrose (fractions #4 to #7), the average size was smaller, 120- 150 nm in both samples (not shown).

**
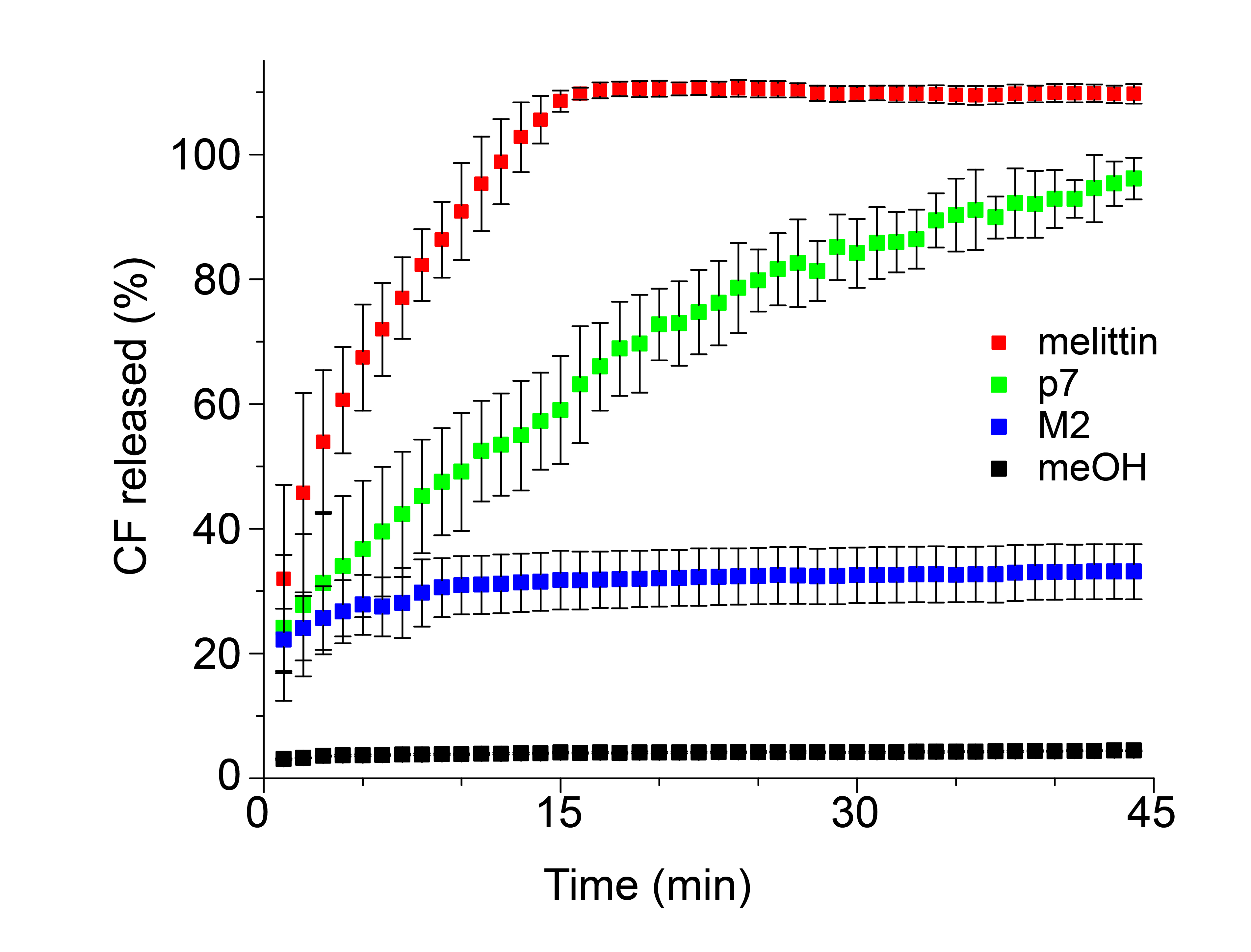
**

**Figure S7. CF release kinetics following addition or extrusion.** (A) Comparison of CF release in the addition method using melittin, p7, M2 (18-60) and only methanol. Details are indicated in the Materials and Methods section of the manuscript.


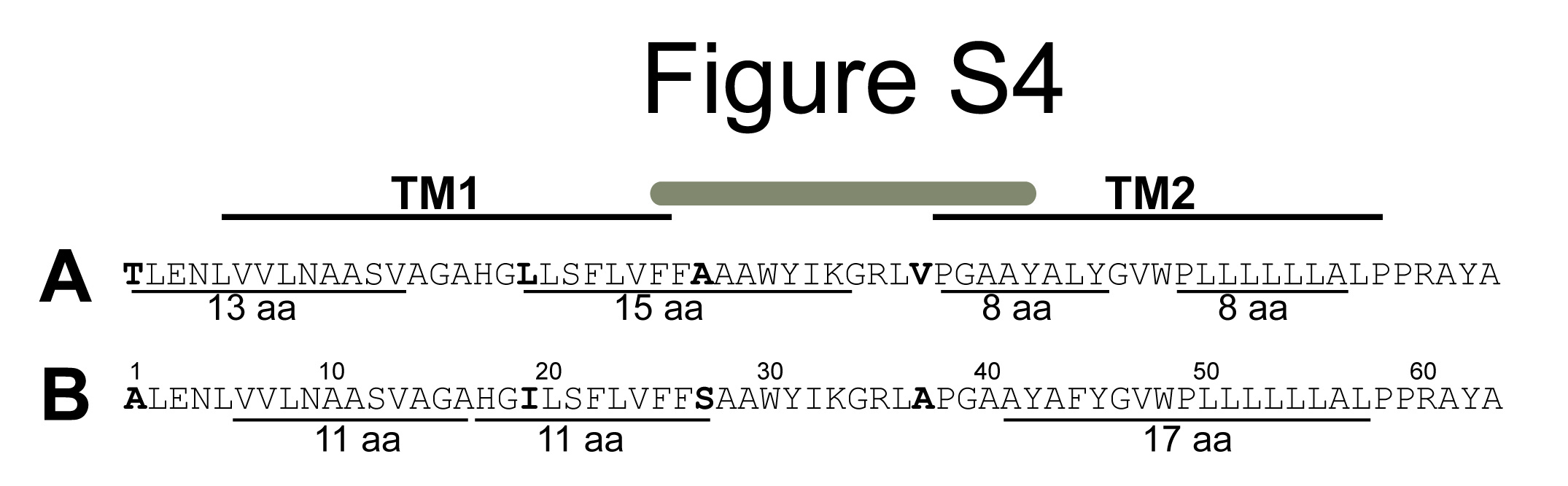


**Figure S8.** Regions of p7 that adopt α-helical conformation according to previous reports. The stretches corresponding to α-helical regions (underlined) reported for p7 in 50% TFE [[1](#_ENREF_1)] (A) and DHPC micelles [[2](#_ENREF_2)] (B), according to NMR experiments. Both sequences correspond to genotype 1b but to different strains, HCV-J (A) and J4 (B). The approximate regions that span TM1 and TM2, and the loop region that produced maximal membrane perturbation[[3](#_ENREF_3)] (grey bar) are indicated. Differences in the sequences are indicated by a bold letter.

1. Montserret R, Saint N, Vanbelle C, Salvay AG, Simorre JP, et al. (2010) NMR structure and ion channel activity of the p7 protein from hepatitis C virus. The Journal of biological chemistry 285: 31446-31461.

2. Cook GA, Opella SJ (2010) Secondary structure, dynamics, and architecture of the p7 membrane protein from hepatitis C virus by NMR spectroscopy. Biochim Biophys Acta 1808: 1448-1453.

3. Perez-Berna AJ, Guillen J, Moreno MR, Bernabeu A, Pabst G, et al. (2008) Identification of the membrane-active regions of hepatitis C virus p7 protein: biophysical characterization of the loop region. J Biol Chem 283: 8089-8101.
